# Thrombotic Thrombocytopenic Purpura in a Patient With Triple‐Negative Breast Cancer Treated With PD‐L1 Inhibition and Taxane Chemotherapy

**DOI:** 10.1002/ccr3.72247

**Published:** 2026-03-12

**Authors:** Jason Ke, Albert C. Chong, Robert Hsu, Anastasia Martynova, Noah Wald‐Dickler, Anishka D'Souza, Caroline Piatek, Gino K. In

**Affiliations:** ^1^ University of Southern California Keck School of Medicine Los Angeles California USA; ^2^ Department of Internal Medicine Mayo Clinic Scottsdale Arizona USA; ^3^ Division of Infectious Disease Los Angeles General Medical Center Los Angeles California USA

**Keywords:** ADAMTS13 protein, atezolizumab, drug‐related side effects and adverse reactions, immune checkpoint inhibitors, thrombotic thrombocytopenic purpura

## Abstract

Thrombocytopenic purpura (TTP) is a hematologic emergency that may occur with PD‐L1 immunotherapy. New or worsening anemia and thrombocytopenia in patients started on PD‐L1 inhibitors should raise suspicion for TTP and prompt workup for thrombotic microangiopathy.

## Graphical abstract

PET imaging of a 56 year old woman with HIV and advanced triple negative breast cancer with regional lymph node and bone metastases.

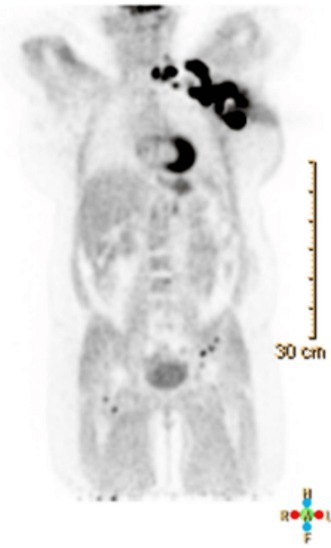

**Abbreviations**
ART: Antiretroviral therapy
BEU: Bethesda equivalent units
DITMA: Drug‐induced thrombotic microangiopathy
INR: International normalized ratio
irAE: Immune‐related adverse events
ITP: Immune thrombocytopenic purpura
PT: Prothrombin time
TMA: thrombotic microangiopathy
TNBC: Triple‐negative breast cancer
TTP: Thrombotic thrombocytopenic purpura
UL‐VWF: Ultra‐large von Willebrand factor

## 1 Introduction

Triple‐negative breast cancer (TNBC) represents 10%–15% of all breast cancers and is characterized by a lack of estrogen receptor, progesterone receptor, and human growth factor receptor 2 [1, 2]. In 2019, the United States Food and Drug Administration approved atezolizumab, an anti‐PDL‐1 immunotherapy, plus nanoparticle albumin‐bound paclitaxel chemotherapy for locally advanced or metastatic TNBC [1, 3]. This regimen demonstrated better response rates (59% vs. 43%) and progression‐free survival (median 7.5 months vs. 5.0 months) than taxane chemotherapy alone, which was the preexisting standard of care [3]. Given that chemoimmunotherapy is now a first‐line treatment for TNBC, there is an urgent need to understand the spectrum of possible complications to improve recognition and optimize management of significant adverse events.

Thrombotic thrombocytopenic purpura (TTP) is a rare hematological emergency with an estimated incidence of 13 per 1 million people in the US per year, and a 90% mortality rate if untreated [4, 5]. TTP occurs when excessive ultra‐large von Willebrand factor (UL‐VWF) multimers in the plasma adhere to platelets, leading to microthrombosis, hemolytic anemia, and thrombocytopenia [6, 7]. Buildup of UL‐VWF is thought to stem from endothelial cell damage and from a deficiency in plasma ADAMTS13, the protease responsible for cleaving UL‐VWF [8, 9]. TTP as an adverse event to PD‐L1 inhibitors is uncommonly reported but highly important [10]. Here, we present a case of an HIV‐positive patient with metastatic TNBC who developed TTP during treatment with atezolizumab and taxane chemoimmunotherapy. This is a unique, educational case where multiple risk factors coincided in a patient who developed TTP.

## 2 Case History/Examination

A 56‐year‐old African American woman presented in January 2021 with a left breast lump that she first noticed 6 months prior. She was diagnosed with HIV in 2016 and had since been on an antiretroviral therapy (ART) regimen of bictegravir/emtricitabine/tenofovir alafenamide. At the time of presentation, her CD4 count was 401 (29.9%) and her viral load continued to be undetectable since 2019. The patient also had a family history of breast cancer in both her mother and maternal grandmother.

**FIGURE 1:**
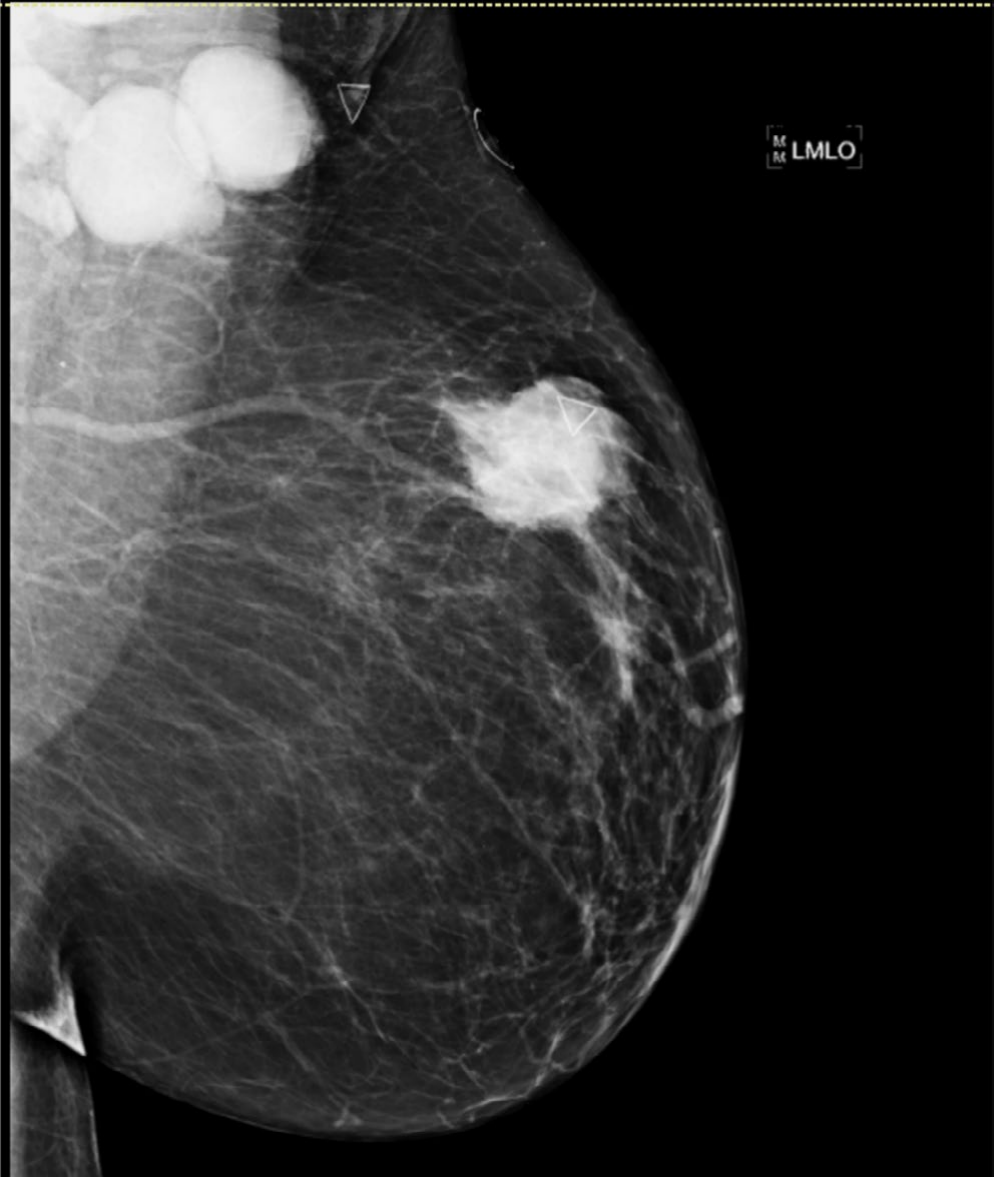
Left breast mass and axillary lymphadenopathy on mammography. Initial mammography showed an irregular mass with spiculated margins in the left breast upper outer quadrant, correlating to a palpable abnormality on clinical breast exam. There is accompanying prominent lymphadenopathy at the left axilla. BI‐RADS 5.

Mammography showed an irregular mass in the upper, outer quadrant of the left breast along with several enlarged lymph nodes in the left axilla (Figure 1).

**FIGURE 2:**
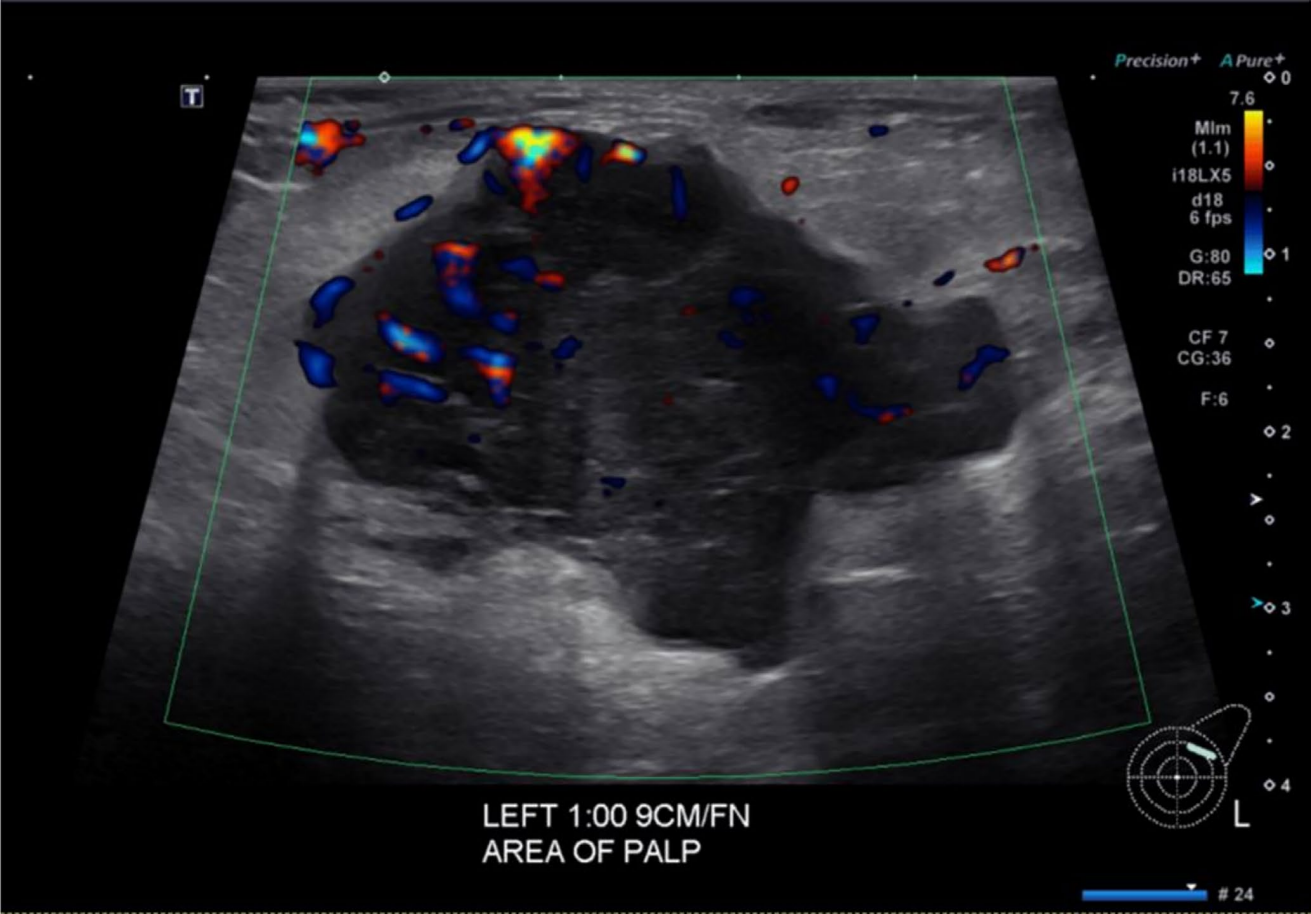
Left breast mass and axillary lymphadenopathy on ultrasound. Ultrasound of the left breast revealed an irregular mass measuring 4.2 × 3.7 × 2.9 cm at the 1 o'clock position, located 9 cm from the nipple, corresponding to the major lesion seen on mammography. Additionally, multiple enlarged lymph nodes were present at the left axilla, demonstrating loss of the normal fatty hilum. BI‐RADS 5.

Ultrasound showed an irregular mass measuring 4.2 × 3.7 × 2.9 cm in the left breast at 1 o'clock, 9 cm away from the nipple, and multiple enlarged left axillary lymph nodes (Figure 2).

**FIGURE 3:**
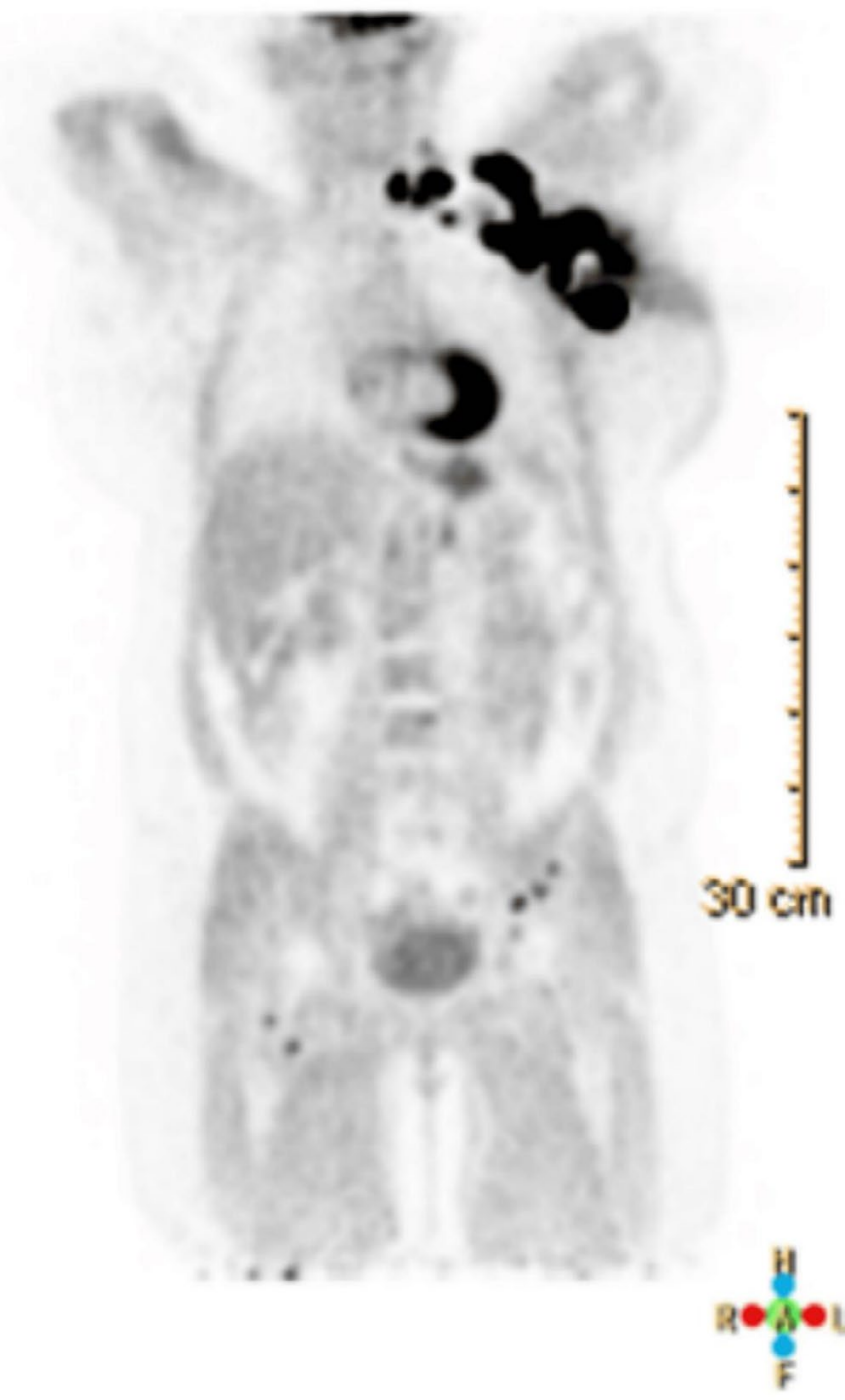
PET/CT scan revealing extensive metastatic disease. PET/CT taken two months after mammogram and ultrasound demonstrated a 4.6 × 4.0 × 5.2 cm hypermetabolic, irregular mass in the left breast, corresponding to the patient's biopsy‐proven invasive ductal carcinoma. Multiple enlarged, irregular, and necrotic‐appearing metastatic lymph nodes were visualized, largest at the left axilla, subpectoral, and infraclavicular regions. Scattered osseous metastases were noted throughout the axial skeleton and proximal bilateral femurs.

Biopsies of the breast and lymph node revealed poorly differentiated ductal carcinoma, ER/PR/HER2 negative. A FDG‐PET scan showed osseous metastases scattered throughout the axial skeleton and proximal bilateral femurs (Figure 3). Given metastatic disease, systemic therapy was initiated in June 2021, with 28‐day cycles, using a combination of atezolizumab 840 mg on Days 1 and 15, and nab‐paclitaxel 100 mg/m^2^ on Days 1, 8, and 15.

During the fourth cycle, on October 17, 2021, the patient experienced sudden fatigue, nausea, and generalized weakness. Serologic studies showed a platelet drop from 182 to 20 × 10^9/L, hemoglobin drop from 12.1 to 5.7 g/dL, and a rise in total bilirubin from 0.5 to 2.4 mg/dL over the past 27 days (Table 1). Lactate dehydrogenase was elevated at 1737 U/L, while haptoglobin was < 10 mg/dL. Peripheral blood smear revealed schistocytes. Direct Coombs test was negative. Coagulation studies revealed a prothrombin time (PT) of 14.7 s with an international normalized ratio (INR) of 1.1. The patient was not on a vitamin K antagonist. ADAMTS13 activity level (via chromogenic ELISA) returned < 3%; reflexive ADAMTS13 inhibitor testing showed 1.4 Bethesda equivalent units (BEU, normal range < 0.4 BEU), together prompting the diagnosis of TTP.

**TABLE 1:** Laboratory results prompting diagnosis of TTP.

Parameter	Result	Normal range
WBC	4.8 × 10^9/L	4.1–10.9 × 10^9/L
RBC	1.9 × 10^9/L	3.8–5.2 × 10^9/L
Hgb	5.7 g/dL	11.0–15.7 g/dL
Hct	16.6%	34.9%–46.9%
MCV	87.6 fL	80–100 fL
RDW	20.4%	11.5%–14.3%
MCH	30.1 pg	26–34 pg
MCHC	34.4 g/dL	31–37 g/dL
Platelet	20 × 10^9/L	150–400 × 10^9/L
MPV	12.7 fL	9.2–12.7 fL
Sodium	140 mmol/L	136–145 mmol/L
Potassium	3.8 mmol/L	3.4–5.2 mmol/L
Chloride	99 mmol/L	98–107 mmol/L
BUN	13 mg/dL	8–23 mg/dL
Creatinine	0.44 mg/dL	0.51–0.95 mg/dL
Calcium	9.4 mg/dL	8.8–10.2 mg/dL
Albumin	3.7 g/dL	3.5–5.2 g/dL
AST	159 U/L	0–35 U/L
ALT	83 U/L	0–35 U/L
Bili total	2.4 mg/dL	< = 1.2 mg/dL
Bili direct	0.8 mg/dL	0.0–0.2 mg/dL
PT	14.7 s	12.3–14.9 s
INR	1.1	0.9–1.1
ADAMTS13 activity assay	< 3%	50%–150%
ADAMTS13 inhibitor assay	1.4 BEU	< 0.4 BEU

## 3 Differential Diagnosis

On presentation, the patient's abrupt decline in hemoglobin and platelets with elevated LDH, diminished haptoglobin, indirect hyperbilirubinemia, and schistocytes on peripheral smear was most consistent with a thrombotic microangiopathy (TMA). Key considerations included immune thrombocytopenic purpura (ITP), drug‐induced thrombotic microangiopathy (DITMA), cancer‐associated microangiopathic hemolytic anemia (MAHA), and TTP. ITP was less likely given the concomitant evidence of hemolysis. Cancer‐associated MAHA and DITMA typically lack severe ADAMTS13 deficiency. Instead, the severe ADAMTS13 deficiency (as indicated by the suppressed ADAMTS13 activity assay) and presence of neutralizing ADAMTS13 antibodies (as indicated by the positive ADAMTS13 inhibitor assay) supported the diagnosis of TTP and guided initiation of appropriate therapy [11]. The timing of this episode in relation to initiation of atezolizumab and nab‐paclitaxel directed us to examine the possibility of an immune checkpoint inhibitor‐related process. Ultimately, repeat ADAMTS13 testing following treatment would demonstrate improved activity levels with persistence at 4 weeks, which in conjunction with the positive ADAMTS13 inhibitor assay, strongly favored immune‐mediated over congenital TTP/Upshaw–Schulman syndrome [11].

## 4 Outcome and Follow‐Up

Chemoimmunotherapy was held and the patient was started on solumedrol 1 g IV daily. The following day, on October 18, 2021, her platelet count dropped further from 20 to 6 × 10^9/L. She received a transfusion of 1 unit of platelets on October 19, with platelets recovering to 84 × 10^9/L. The patient was then started on daily plasmapheresis. On October 20, the platelet count was 67 × 10^9/L; she was started on rituximab 1 g IV every 2 weeks and switched from solumedrol to oral prednisone 70 mg daily. By October 22, the platelets had declined to 27 × 10^9/L. Follow‐up CT scans on October 26 demonstrated stability of the large left breast mass and associated lymphadenopathy, as well as a new moderate left pleural effusion with left basilar pleural nodularity concerning for pulmonary metastases.

Platelets gradually rose to greater than 50 × 10^9/L by October 27, 8 days after starting plasmapheresis, and remained stable in the range of 50–60 × 10^9/L thereafter. Given stabilization of the platelet count, daily plasmapheresis was stopped on November 13, after a total of 25 days. Posttreatment ADAMTS13 activity level on November 14 returned at 83%, and platelets remained in the 50–60 × 10^9/L range. The persistent thrombocytopenia was attributed to infiltration of cancer into the bone marrow, as supported by early myeloid and erythroid cells detected on the peripheral smear, though no biopsy was done. She was discharged home on December 15 on prednisone 70 mg PO daily. She was readmitted 2 days later for cellulitis of the right lower extremity, for which she was given clindamycin and doxycycline. Her platelet count was 77 × 10^9/L at the time of readmission, and ADAMTS13 activity level returned at 53% (remission phase at 1 month posttreatment). Follow‐up studies also showed a PT of 13.9 s with INR 1.08. The patient was discharged on December 19th and was subsequently lost to follow‐up. She expired 2 months later without a documented cause of death.

## 5 Discussion

TTP is a disease of imbalance between UL‐VWF and its protease ADAMTS13. This case features a confluence of risk factors for TTP, namely TNBC, HIV positivity, and chemoimmunotherapy [4, 12, 13, 14]. While ADAMTS13 deficiency was serologically evident in our patient, there is no clear mechanism by which any of the aforementioned risk factors directly cause deficiency of this protease [12, 14, 15, 16]. Instead, we propose that progressing malignancy and chronic HIV contributed to increased circulatory UL‐VWF through the mechanism of endothelial cell stimulation, damage, or apoptosis [8, 9, 13, 17]. We hypothesize that chemoimmunotherapy then lowered functional ADAMTS13 activity below a critical threshold, thereby triggering the clinical manifestation of TTP.

Among cancer patients, TMAs have an annual incidence rate of 0.3 to 0.5 cases per million [14, 16, 18, 19]. Cancer is thought to cause TMAs via tumor invasion of the bone marrow or vascular obstruction, leading to erythrocyte fragmentation and platelet consumption [14, 16, 20]. However, cancer‐based TMAs do not exhibit severe ADAMTS13 deficiency at presentation and are poorly responsive to plasma exchange [6, 15, 16, 18, 19], making it unlikely to be the cause of our patient's TTP. Alternatively, it is possible that cancer contributed to the onset of TTP by way of UL‐VWF accumulation. Multiple cancer types, including breast cancer, may induce UL‐VWF secretion from endothelial cells or release it directly from tumors, resulting in significantly elevated plasma UL‐VWF levels [21, 22, 23, 24].

To our knowledge, nab‐paclitaxel has not been associated with TTP in the medical literature and taxanes are rarely associated with TMAs [16, 25, 26]. Cytotoxic chemotherapy can lead to DITMA through an unknown mechanism, with the most frequently implicated agents being gemcitabine and mitomycin [26]. However, the presentation of DITMA differs from classical TTP. DITMAs are strongly correlated with kidney damage, which our patient did not exhibit, and they are not associated with ADAMTS13 deficiency, nor effectively treated with plasma exchange [16, 26]. Due to these inconsistencies, and without clear evidence in the literature to support nab‐paclitaxel triggering UL‐VWF release from the endothelium or diminishing of ADAMTS13 activity levels, it is unclear if there was any association between our patient's chemotherapy and her TTP.

On the other hand, TTP is a well‐established complication of HIV: the incidence of HIV‐associated TTP has been estimated at 18 to 64 cases per million compared to 2 to 6 cases per million for TTP alone [4, 12, 27, 28, 29]. The exact pathophysiology of HIV‐induced TTP is unknown, though it is unlikely to be driven by autoimmunity [12, 29]. Meanwhile, vascular disease is a known complication of HIV, including for patients on ART [12, 30, 31]. HIV proteins, such as transactivator of transcription, negative factor, and membrane glycoproteins, may cause endothelial inflammation and vascular disease, with subsequent elevation of UL‐VWF levels [12, 27, 32].

While malignancy, HIV, and possibly chemotherapy can each contribute to TTP, we suspect atezolizumab played a significant role. Rare case reports describe TTP in patients treated with immune‐checkpoint inhibitors, most commonly nivolumab but also several cases associated with atezolizumab [33, 34, 35, 36, 37, 38, 39]. TTP onset ranges from a few days after the first cycle to occurring after the eighth cycle, 6 months into treatment [33, 40]. Anti‐PD‐L1 drugs have been reported in association with immune‐related adverse events (irAEs), most commonly affecting the endocrine, gastrointestinal, cardiac, and musculoskeletal systems [41, 42, 43, 44, 45]. Hematological complications of anti‐PD‐L1 drugs are less common, comprising roughly 3%–4% of total irAEs, with the most common being cytopenias, including anemia and thrombocytopenia [44]. Immune checkpoint inhibitors including PD‐L1 inhibitors are known to trigger formation of de novo autoantibodies or increase preexisting autoantibody titers, which is mechanistically linked to the pathogenesis of irAEs [46, 47, 48]. Thus, it is plausible that PD‐L1 inhibitors may trigger TTP via development of ADAMTS13 antibodies although this has not been fully investigated in the literature.

While we caution against overinterpretation of our findings, the severe deficiency of ADAMTS13 and confirmed presence of ADAMTS13 antibodies in this case presents an example of a patient where TTP developed following treatment with PD‐L1 inhibition‐based chemoimmunotherapy. Certainly, the role of chemoimmunotherapy as the trigger for this case of TTP cannot be definitively proven, particularly given the multitude of other risk factors. Fortunately, it is worth noting that regardless of the inciting event, this medically complex patient did still respond to standard therapeutic approaches for TTP, including corticosteroids, plasma exchange, and rituximab. As more cancer patients are treated with immune checkpoint inhibitors going forward, we believe that further investigation into the potential for autoimmune hematologic events secondary to these drugs warrants further investigation.

In conclusion, new or worsening anemia and thrombocytopenia in a patient undergoing treatment with PD‐L1 immunotherapy should raise suspicion for TTP, especially when accompanied by risk factors such as malignancy, infections, and chemotherapy that may reduce the threshold for TTP via the mechanism of endothelial cell damage and UL‐VWF release. With greater utilization of checkpoint inhibitors across multiple cancer types [49], our report highlights the need for a more robust understanding of TTP pathophysiology as it relates to medications and comorbidities for safer and more informed clinical practice.

## Author Contributions

**Jason Ke:** investigation, writing – original draft, writing – review and editing. **Albert C. Chong:** data curation, investigation, writing – original draft, writing – review and editing. **Robert Hsu:** supervision, writing – review and editing. **Anastasia Martynova:** supervision, writing – review and editing. **Noah Wald‐Dickler:** supervision, writing – review and editing. **Anishka D'Souza:** supervision, writing – review and editing. **Caroline Piatek:** data curation, supervision, writing – review and editing. **Gino K. In:** conceptualization, data curation, investigation, supervision, writing – review and editing.

## Funding

The authors have nothing to report.

## Consent

The patient was deceased at the time of study. Written informed consent was obtained from the patient's next‐of‐kin to publish this report in accordance with the journal's patient consent policy.

## Conflicts of Interest

The authors declare no conflicts of interest.

## Data Availability

Data sharing not applicable to this article as no datasets were generated or analyzed during the current study.
